# Parvovirus-Based Combinatorial Immunotherapy: A Reinforced Therapeutic Strategy against Poor-Prognosis Solid Cancers

**DOI:** 10.3390/cancers13020342

**Published:** 2021-01-19

**Authors:** Assia Angelova, Tiago Ferreira, Clemens Bretscher, Jean Rommelaere, Antonio Marchini

**Affiliations:** 1 German Cancer Research Center (DKFZ), Research Program Infection, Inflammation and Cancer, Clinical Cooperation Unit Virotherapy, Im Neuenheimer Feld 242, 69120 Heidelberg, Germany; j.rommelaere@dkfz-heidelberg.de; 2 German Cancer Research Center (DKFZ), Laboratory of Oncolytic-Virus-Immunotherapeutics (LOVIT), Im Neuenheimer Feld 242, 69120 Heidelberg, Germany; t.ferreira@dkfz-heidelberg.de (T.F.); c.bretscher@gmx.de (C.B.); antonio.marchini@lih.lu (A.M.); 3 Luxembourg Institute of Health (LIH), Laboratory of Oncolytic-Virus-Immunotherapeutics (LOVIT), 84 rue Val Fleuri, L-1526 Luxembourg, Luxembourg

**Keywords:** parvovirus, oncolytic, tumor microenvironment, immunotherapy, combination therapy, glioblastoma, pancreatic cancer, colorectal cancer, melanoma

## Abstract

**Simple Summary:**

Oncolytic virotherapy using oncolytic viruses with natural or engineered cancer-destroying capacities has emerged as a promising treatment concept in modern oncology. Rodent protoparvoviruses, in particular the rat H-1 parvovirus (H-1PV), have demonstrated their broad-range tumor-suppressive properties in both preclinical models and clinical studies. In addition to inducing selective tumor cell death, these viruses are also able to exert immunostimulating effects and reverse tumor-driven immune suppression. Parvovirotherapy holds therefore a potential for enhancing the efficacy of other cancer immunotherapies. The aim of this review is to provide an overview of all H-1PV-based combinatorial immunotherapeutic approaches against poor-prognosis human solid cancers that have been tested so far. Current challenges and future prospects of parvoviro-immunotherapy, notably parvovirus inclusion into various immunotherapeutic protocols against glioblastoma, pancreatic cancer, among other standard therapy-refractory solid malignancies, are also discussed in the light of H-1PV further clinical development.

**Abstract:**

Resistance to anticancer treatments poses continuing challenges to oncology researchers and clinicians. The underlying mechanisms are complex and multifactorial. However, the immunologically “cold” tumor microenvironment (TME) has recently emerged as one of the critical players in cancer progression and therapeutic resistance. Therefore, TME modulation through induction of an immunological switch towards inflammation (“warming up”) is among the leading approaches in modern oncology. Oncolytic viruses (OVs) are seen today not merely as tumor cell-killing (oncolytic) agents, but also as cancer therapeutics with multimodal antitumor action. Due to their intrinsic or engineered capacity for overcoming immune escape mechanisms, warming up the TME and promoting antitumor immune responses, OVs hold the potential for creating a proinflammatory background, which may in turn facilitate the action of other (immunomodulating) drugs. The latter provides the basis for the development of OV-based immunostimulatory anticancer combinations. This review deals with the smallest among all OVs, the H-1 parvovirus (H-1PV), and focuses on H-1PV-based combinatorial approaches, whose efficiency has been proven in preclinical and/or clinical settings. Special focus is given to cancer types with the most devastating impact on life expectancy that urgently call for novel therapies.

## 1. Introduction

The rodent H-1 protoparvovirus (H-1PV) (for an overview of H-1PV classification and biology, we redirect the readers to a recent review by Bretscher and Marchini [1]) was first discovered as a contaminating agent in xeno-transplanted human tumor cell lines [2]. Originally identified as a pathogen, which lethally affects rat fetuses and newborn rats by causing cerebellar hypoplasia and hepatitis [3], H-1PV was later found to preferentially replicate in rat- and in human-transformed or tumor-derived cell cultures, while sparing their non-malignant counterparts [4,5]. H-1PV intrinsic oncotropism and oncoselectivity are a complex phenomenon based on multiple molecular determinants, which are underrepresented in normal cells, but characteristic of tumor cells [6]. Importantly, humans are not naturally infected with this virus, and no association between H-1PV and human disease has been observed [7]. Two early clinical studies of virus administration to cancer patients—dating back to the 1960s and 1990s of last century—demonstrated the lack of H-1PV pathogenic effects and the feasibility of the approach [8,9], thus laying the groundwork for the development of parvovirus (PV)-based oncolytic virotherapy. Three decades of laboratory efforts brought about extensive preclinical evidence of H-1PV broad tumor-suppressive potential [5,10]. Furthermore, it became increasingly apparent that in addition to directly inducing cancer cell death (oncolysis), H-1PV was also capable of exerting immuno-stimulating effects in various preclinical cancer models [11,12].

**Figure 1:**
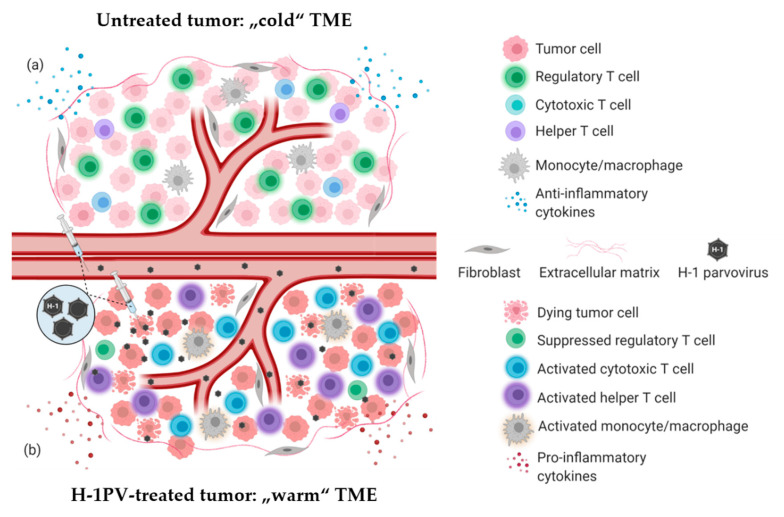
H-1PV-induced modulation of tumor microenvironment immune landscape. (**a**) Immunosuppressive (“cold”) tumor microenvironment (TME) of a solid tumor. The tumor is often infiltrated with abundant immunosuppressive regulatory T cells (Treg)/myeloid-derived suppressor cells (MDSC). Tumor-infiltrating lymphocytes (TILs) (CD8+ CTLs, CD4+ Th cells) are scarce and/or anergic. Tumor and various TME cells produce anti-inflammatory cytokines to maintain immune suppression and facilitate tumor growth and dissemination. (**b**) Tumor infection with H-1PV results in immunogenic tumor cell death leading to the release proinflammatory cytokines, pathogen- and danger-associated molecular patterns (PAMPs and DAMPs), which alarm the immune system. The infection of tumor cells does not necessarily have to be productive for this immuno-stimulating effect to be achieved. Furthermore, abortive infection of immunocytes (CTLs, Th cells, monocytes/macrophages) with H-1PV can also lead to their activation. In contrast, H-1PV inhibits the immune suppressive functions of Treg cells. An immunological switch takes place and converts the “cold” TME into a “warmed up” (inflamed) one. Virus-mediated immuno-conversion of TME favors the mounting of enhanced antitumor immune responses.

PV induced immune system stimulation results from multiple infection-associated immunogenic events. Depending on the tumor model, virus dose, route of administration and the immunological status of the host, one or another immunogenic stimulus may prevail [11]. Regardless of the particular mechanism involved, PV-mediated immunomodulation contributes to the “warming up” of the tumor microenvironment (TME) (Figure 1), increases tumor visibility and enhances immune cell reactivity [13]. H-1PV infection-associated immunogenic events and their impact on the immune system are reviewed in detail elsewhere [12,13], and briefly summarized below.

- Immunogenic cell death (ICD) of H-1PV-infected tumor cells (indirect immune cell stimulation): PVs are potent triggers of immunogenic stimuli through tumor cell ICD induction. Infected tumor cells release a spectrum of proinflammatory mediators, in particular chemo- and cyto-kines, and pathogen- and danger-associated molecular patterns (PAMPs, DAMPs), which are in turn capable of boosting the maturation and reactivity of distinct immune cell populations. This can be exemplified by H-1PV-infected human melanoma cells, which activate dendritic cell (DC) maturation through the release of heat shock protein 72 [14]. In line with this observation, H-1PV-infected pancreatic and colorectal carcinoma cells were shown to stimulate natural killer (NK) cell tumor-killing capacity through both the overexpression of ligands specific for NK cell activation receptors and the downregulation of MHC I on infected tumor cells [15,16]. Notably, productive infection of tumor cells is not required for immune stimulation. This was demonstrated by co-incubating H-1PV-infected semi-permissive pancreatic carcinoma cells with peripheral blood mononuclear cells (PBMC), under which conditions induction of Th1 signature and release of interferon-gamma (IFN-γ) and tumor necrosis factor-alpha (TNF-α) were detected in the PBMC population [17].
- H-1PV infection of immune cells (direct immune cell stimulation): H-1PV infection of human immune cell subpopulations has been documented in various preclinical settings. Virus entry may take place in T, B, NK, DC and monocytic populations; however, infection is aborted at subsequent virus intracellular replication steps [18]. Abortive infection can nevertheless exert multiple immuno-stimulating effects, such as expression of IFN-stimulated genes and proinflammatory cytokine production [17,18]. On the other hand, H-1PV is able to inhibit the immune suppressive activity of regulatory T (Treg) cells [18].
- H-1PV impact on tumor vasculature: It has been demonstrated that endothelial (precursor) cells may constitute direct targets for parvovirus-mediated toxicity. These cells sustain an abortive H-1PV infection in vitro. In animal models, virus treatment inhibits the growth of lymphatic endothelium-derived tumors (Kaposi’s sarcoma). Furthermore, recombinant propagation-deficient parvoviral vectors armed with angiostatic chemokines achieve significant reduction of vascular endothelial growth factor (VEGF) expression in Kaposi’s sarcoma cells [19]. Given the control exerted by the vasculature of tumors over their infiltration with immune cells, these effects are likely to contribute to H-1PV immuno-stimulating activity, as further discussed below. Altogether, these data warrant validation of H-1PV as a tool against highly vascularized cancers, e.g., glioblastoma, one of the most angiogenic human tumors.

The above-outlined H-1PV potential for creating a proinflammatory immune environment and alerting the immune system to the presence of a tumor opens prospects for combining the virus with various immunomodulators or other therapeutic agents endowed with immuno-stimulating properties. This combinatorial approach is in particular promising for the treatment of human tumors that remain presently incurable and pose continuing research and clinical challenges. Pancreatic ductal adenocarcinoma (PDAC), glioblastoma, colorectal cancer (CRC) and melanoma are among those cancers, which are urgently calling for novel therapeutic strategies. H-1PV-based immunotherapeutic combinations are reviewed below, which aim at targeting these devastating malignancies.

## 2. Parvovirus-Based Combinatorial Immunotherapy against Pancreatic Cancer

PDAC is the most common neoplasm of the pancreas and one of the most aggressive human cancers. It is characterized by quick progression, broad intraperitoneal dissemination (peritoneal carcinomatosis) and frequent resistance to conventional treatments. PDAC is usually diagnosed at advanced stages, when surgical resection is either not feasible or inefficient, as most patients eventually suffer from local recurrence and metachronous metastasis [20]. Current chemotherapy regimens achieve only minor improvements of PDAC dismal prognosis: the median survival time and overall 5-year survival remain as low as <12 months and approximately 5%, respectively [21]. Gemzar (gemcitabine) is the standard drug used to treat PDAC patients after surgery. Yet, gemcitabine only prolongs the survival of the majority (82%) of the patients by less than two-fold. On the same line, pathway-specific targeted therapies showed little efficacy against PDAC [22]. Therefore, new treatment paradigms need to be urgently explored in order to extend PDAC patient life expectancy and offer better quality of life.

H-1PV is among the oncolytic viruses (OVs), which have promising potential for efficiently targeting pancreatic cancer. PDAC sensitivity to H-1PV-induced oncolysis was demonstrated in various preclinical models [23,24]. Infection of human PDAC-derived cells leads to their killing, which is mediated at least in part by cathepsins [24]. Importantly, H-1PV sensitivity is preserved in gemcitabine-resistant cultures [23], thus opening up prospects to circumvent PDAC resistance to current standard death inducers.

### 2.1. H-1PV + Nucleoside Analogues (Gemcitabine)

As gemcitabine is currently considered the gold chemotherapeutic standard in PDAC clinical management, the therapeutic efficacy of gemcitabine in combination with H-1PV was tested in a rat syngeneic orthotopic PDAC model. H-1PV administration to gemcitabine-pretreated animals led to significant tumor suppression and survival prolongation in comparison with the mock-infected or gemcitabine-only treated groups [23]. These in vivo findings could not be straightforwardly ascribed to synergistic tumor cell death enhancement only. Indeed, in vitro studies showed that the cytotoxic effects of the combination, while allowing effective dose reduction for both agents, did not result in complete PDAC culture elimination. This prompted the investigation of the immunological effects exerted by the H-1PV + gemcitabine combination as an added value to direct tumor cell killing. Markers of ICD induction were analyzed in various PDAC cell lines, treated with either virus (or gemcitabine) alone or with H-1PV + gemcitabine. It was demonstrated that the release of high-mobility group box 1 protein (HMGB1) is a strikingly robust feature of H-1PV-infected PDAC cells [24]. Furthermore, H-1PV-triggered HMGB1 release did not require lytic infection, in line with the above-described PBMC activation by non-productively infected PDAC cells [17]. Gemcitabine alone was unable to induce HMGB1 secretion, yet H-1PV-induced HMGB1 release remained unaffected in gemcitabine-treated cells. Gemcitabine, on the other hand, was able to induce—albeit not in all cell lines tested—mature interleukin 1-beta (IL-1ß) accumulation in culture supernatants. Taken together, these data show that H-1PV and gemcitabine complement each other in the induction of immunogenic signals. The compatibility of H-1PV-induced alarmin (HMGB1) secretion with other (ICD-inducing) chemotherapeutic regimens warrants the consideration of PV inclusion into various multimodal anti-PDAC treatment protocols [24]. The therapeutic promise of H-1PV administration in gemcitabine-treated pancreatic cancer patients is further supported by several reports in the literature showing that, unlike most nucleoside analogues, gemcitabine is lacking immunosuppressive properties. On the contrary, gemcitabine may be beneficial not only to the cytocidal but also to the pro-immune outcome of H-1PV infection, as assumed from the findings below.

- One study conducted in gemcitabine-treated PDAC patients revealed the ability of the drug to enhance T cell-mediated and DC-dependent host immune responses [25].
- In keeping with the aforementioned data, it was documented that gemcitabine therapy may promote naïve T cell activation in PDAC patients and enhance their responsiveness to specific vaccination or to other forms of immunotherapy [26].
- The understanding of gemcitabine immunoregulating effects as a complementary constituent of tumor cell toxicity was extended by the demonstration that this drug alleviates pancreatic cancer immune escape through NK cell cytotoxicity enhancement [27].
- Studies conducted in murine orthotopic PDAC models provided yet another insight into gemcitabine-mediated immuno-stimulation, namely by indicating that low chemotherapeutic doses selectively deplete effector/memory Treg cell populations. The latter has a strong impact on PDAC microenvironment, as Tregs usually form large intra-tumoral infiltrates and trigger local immune suppression [28,29].
- Last but not least, in cancer models other than PDAC, gemcitabine enhances the efficacy of OV (e.g., reovirus) therapy. This complementation is achieved through gemcitabine-mediated inhibition of myeloid-derived suppressor cell (MDSC) recruitment to the TME and acceleration of reovirus-induced antitumor T cell immune responses [30].

Based on favorable preclinical data hinting at the potentiation of OV-induced antitumor effects in the presence of gemcitabine, a clinical trial, ParvOryx02 (NCT02653313), was designed and conducted with the aim to provide a clinical proof-of-principle of the safety (and efficacy) of H-1PV + gemcitabine co-treatment. Patients with inoperable metastatic (at least one hepatic metastasis) pancreatic cancer were treated with H-1PV. The virus was first administered intravenously (40% of the total virus dose on four consecutive days), and the remaining virus dose was then given intra-metastatically as single hepatic injection, followed by gemcitabine treatment [31]. Partial response and extended overall survival were observed in two out of seven trial patients, and immunological signatures most likely contributed to this improved outcome. The ParvOryx02 study therefore provided the first clinical indication that immune mechanisms underlie PV-mediated tumor suppression [32].

### 2.2. H-1PV + Histone Deacetylase Inhibitors (Valproic Acid)

Preclinical proof-of-concept was also obtained for another treatment combining H-1PV with the histone deacetylase (HDAC) inhibitor (HDACi) valproic acid (VPA) [33]. HDACis hold significant promise in cancer therapy, due to their ability to cause malignant cell growth inhibition, re-differentiation and death [34]. Most interestingly, HDAC inhibition was also found to potentiate the oncotoxicity of various OVs, including vesicular stomatitis [35], herpes- [36], adeno- [37] and parvo [33]-viruses (for a review, see Reference [38]). The synergism between HDACi and H-1PV was first demonstrated by Li et al., who conducted preclinical testing of this combination in cervical carcinoma and PDAC models [33]. VPA proved to synergize with H-1PV in inducing DNA damage, oxidative stress and death in PDAC-derived cell lines. This cooperation was traced back, at least in part, to the ability of VPA to stimulate the acetylation and, in consequence, the oncotoxic activity of the viral protein NS1. Interestingly, VPA-induced hyperacetylation of NS1 was also associated with enhanced H-1PV DNA replication and viral gene transcription, ultimately boosting virus multiplication in tumor cells. The VPA-dependent increase in both H-1PV intrinsic oncotoxicity and multiplication was reflected in the potentiation of tumor suppression in animal models. In order to establish a clinically relevant animal model of PDAC, patient-derived material was xeno-transplanted in non-obese diabetic/severe combined immunodeficiency disease (NOD/SCID) mice. Alternatively, the human AsPC-1 cell line was implanted into nude rats. Tumors were subjected to mono versus combinatorial treatment and tumor growth parameters were comparatively evaluated. In line with the in vitro observations, H-1PV + VPA administration resulted in enhanced NS1 and H-1PV intra-tumoral accumulation, correlating with an increase in oxidative stress and subsequent apoptosis in co-treated tumors. The combination achieved complete AsPC-1 tumor eradication. Patient-derived xenografts were also responsive, yet to a somewhat lesser extent, probably due to the characteristic PDAC intra-tumoral heterogeneity and prominent presence of stroma.

It is noteworthy that besides its effects on tumor cell growth and OV oncotoxicity, VPA was reported to modulate the immune system, providing an additional possible interface for cooperation with OVs at the level of their intrinsic immuno-stimulating activity. VPA was indeed shown to:

- Exert epigenetic regulation of various immune functions, e.g., attenuation of MDSC immunosuppressive effects [39].
- Induce the expression of MHC I-related chain A (MICA) and B (MICB) molecules, as well as of UL16-binding proteins (ULBPs) in human tumor cells, thereby triggering their enhanced recognition by NK cells [40], like H-1PV does (see above [15,16]).
- Mediate the inhibition of macrophage migration inhibitory factor (MIF) expression through local chromatin deacetylation-based transcription targeting [41].

As a whole, the above data speak for the high translational relevance of VPA to the future development of PV-based combinatorial (immuno) therapies.

**Figure 2:**
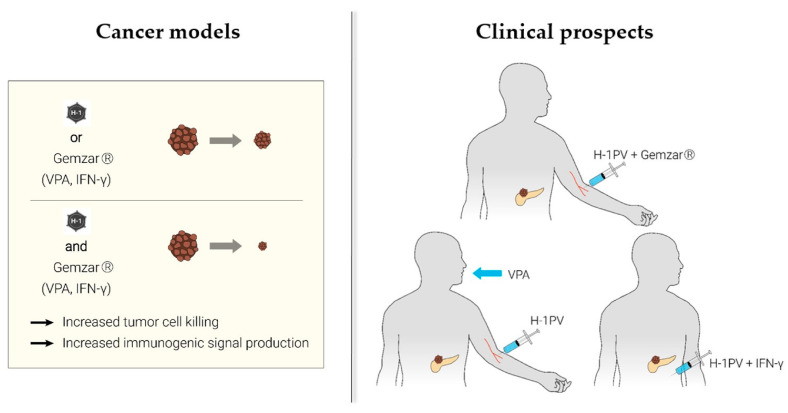
Parvovirus-based viro-immunotherapeutic combinations under development against pancreatic ducatl adenocarcinoma (PDAC). H-1PV-induced tumor cell lysis cooperates with gemcitabine-triggered programmed tumor cell death, valproic acid (VPA)-dependent epigenetic transcription regulation or interferon (IFN)-γ-induced immuno-stimulation to suppress PDAC. Preclinical data suggest that the immune system mediates, at least in part, this cooperation. H-1PV infection of tumor cells leads to the release of PAMPs/DAMPs, such as high-mobility group box 1 protein (HMGB1), which in turn alert the immune system to danger and mobilize an inflammatory antitumor immune response. Various aspects of H-1PV-, gemcitabine-, VPA- and IFN-γ-exerted immunomodulation may converge and synergize upon exposure of the host immune system to the respective combinations. The underlying mechanisms remain to be elucidated in detail by gathering extensive clinical experience. For details and references, see main text.

In conclusion, two drugs that are available on the pharmaceutical market, i.e., gemcitabine (cytostatic) and VPA (antiepileptic), proved to be efficient in synergizing with H-1PV to suppress pancreatic cancer (Figure 2).

### 2.3. H-1PV + Proinflammatory Cytokines (Interferon-Gamma)

Another combination with substantial potential for clinical development relies on the mutual complementation of H-1PV- and IFN-γ-mediated immune stimulation. It was shown that IFN-γ improves the vaccination potential of the virus and diminishes the development of peritoneal carcinomatosis in preclinical PDAC models. Concomitant intraperitoneal administration of both H-1PV and IFN-γ in these models led to extended animal survival correlating with enhanced peritoneal macrophage and splenocyte responses against tumor cells [42].

## 3. Parvovirus-Based Combinatorial Immunotherapy against Glioblastoma

Glioblastoma multiforme (GBM) is the most common and aggressive human primary brain tumor. Similar to PDAC, GBM patients experience a very poor outcome. The 5-year overall survival rate is very low, around 5.1% [43]. GBM treatment faces a unique challenge: the presence of the blood–brain barrier (BBB), which largely prevents drugs, including small-molecule ones, from entering the central nervous system [44]. Current therapeutic approaches therefore include surgical resection of the tumor—to the largest extent feasible and safe—followed by radiotherapy and concomitant chemotherapy [45]. Unfortunately, despite all clinical efforts, tumor progression and recurrence typically occur, calling for alternative therapeutic solutions [46].

Based on the so far unmet need for novel, more efficient treatments, GBM was among the preclinical tumor models most extensively studied in our laboratory. H-1PV capacity for selectively killing glioma cells through cytosolic activation of lysosomal proteases was first demonstrated in vitro [47]. These results were validated in animal models, namely in immunocompetent rats bearing orthotopic autologous RG-2 tumors and in immunodeficient rats bearing xeno-transplanted human U87 gliomas. In these models, tumor regression after local, intravenous or intranasal virus administration was observed [47,48,49]. H-1PV treatment was not associated with any significant off-target toxicities; accordingly, virus transcription and NS1 protein accumulation could be detected in regressing tumor remnants and not in the surrounding normal tissues [48]. Interestingly, the therapeutic effect was potentiated in the presence of an intact host immune system. T cell depletion impaired H-1PV-induced glioma suppression; conversely, the presence of T cell only, in the absence of PV treatment, was not sufficient to inhibit tumor growth [11]. These preclinical observations provided the first hints of host T cell response involvement in PV-mediated glioma regression, hence the rationale for the development of PV-based immunotherapies against glioblastoma.

Pursuant to the above-described preclinical findings, the ParvOryx01 trial (NCT01301430) in recurrent glioblastoma patients delivered the first clinical proof-of-concept for tumor-infiltrating lymphocytes (TILs) playing substantial role in H-1PV-mediated immunomodulation of GBM TME. Although ParvOryx01 primary objectives were to determine virus safety, tolerability, pharmacokinetics, shedding and maximum tolerated dose, the analysis of post-virus-treatment resected tumor tissues revealed the presence of prominent immune cell infiltrates [50]. These infiltrates were comprised of CD45+CD3+CD4+ and CD45+CD3+CD8+ TILs. The latter contained both perforin and granzyme B-positive secretory granules, which is indicative of CTL cytolytic activity. TILs proved, in addition, to be CD25 (IL2 receptor alpha chain)-positive. Only a minor fraction of these cells expressed FOXP3, indicating the scarcity of Treg cells within the intra-tumoral immune infiltrates. Intra-tumoral production of proinflammatory cytokines (IFN-γ, IL-2) was also detected, together with inducible nitric oxide synthase (iNOS) expression in CD68+ tumor-associated microglia/macrophage cells [50,51]. Interestingly, tumor cells expressed the CD40 ligand (CD40L), a positive prognostic factor in glioblastoma [52]. Co-expression of CD40L and CD40, considered as a negative prognostic factor, was not seen [50,51]. Taken together, these first clinical findings indicated that H-1PV has the capacity to exert immuno-stimulating effects on glioblastoma TME. This makes the virus a worthwhile partner in therapeutic combinations, which aim at warming up the intrinsically immunosuppressive and immune-evasive environment of brain tumors.

### 3.1. H-1PV + Ionizing Radiation

We have previously shown that radiotherapy, one of the conventional first-line treatments in glioblastoma patients, sensitizes low-passage glioma cultures to H-1PV oncolysis. Pre-irradiation increases the susceptibility of these cells to virus infection. Interestingly, H-1PV achieves killing both radiation-sensitive and resistant glioma cells [53]. Apart from triggering enhanced tumor cytolysis, the irradiation followed by H-1PV treatment holds, in addition, the potential—although not yet validated in animal models—of acting as combinatorial immunotherapy. Indeed, although irradiation was long regarded as a local anticancer therapy, the first reports on radiotherapy interactions with the host immune system can be traced back to the 1970s of the last century. In 1979, Slone et al. were the first to report that the radiation dose required to control 50% of mouse fibrosarcomas was twice as high in immunocompromised animals as in immunocompetent hosts [54]. Furthermore, tumor regression at sites distant to radiation fields, the so-called abscopal effect, has been systematically observed [55]. Radiation-triggered immunomodulation encompasses, among other effects, ICD induction, T and NK cell activation and MDSC suppression. These observations prompted the development of various combination therapy regimens based on radiation and other immunomodulating agents [56,57], including OVs (e.g., adeno-, herpes simplex-, measles- and vaccinia-viruses) against glioma [58].

### 3.2. H-1PV + Tumor Angiogenesis Inhibitors (Bevacizumab)

**Figure 3:**
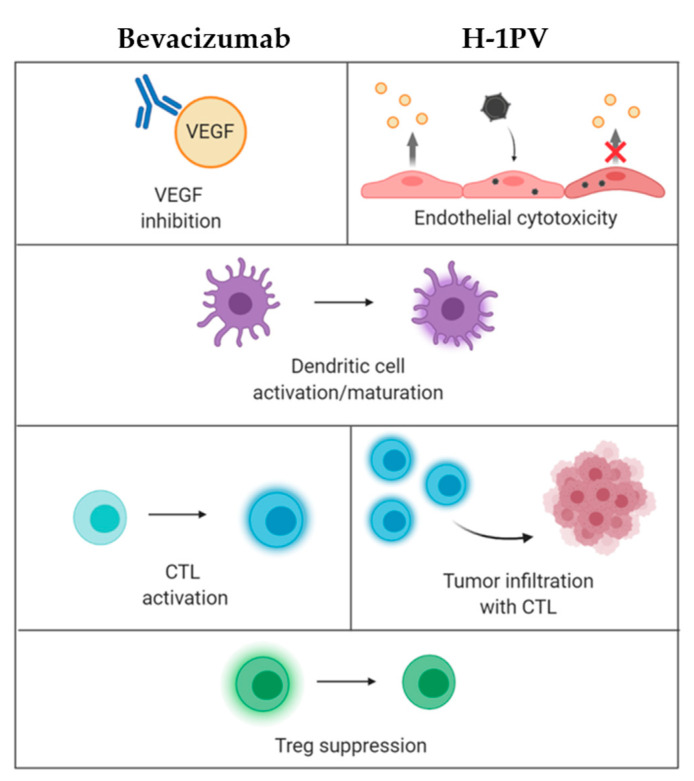
Rationale for combining H-1PV administration with bevacizumab treatment in patients with recurrent glioblastoma multiforme (GBM). Bevacizumab antibody and H-1PV infection share the capacity for inhibiting vascular endothelial growth factor (VEGF) (production) (upper row) and triggering distinct immuno-modulations (lower rows), raising hopes to improve antitumor immunity by combining both treatments. In support of this strategy, bevacizumab and H-1PV were found to jointly achieve significant clinical improvement in GBM patients at second or third recurrence, leading to remission of the recurrent tumor. The precise mechanisms of this therapeutic potentiation remain to be determined. However, the establishment by H-1PV of an immunologically “improved” proinflammatory background, which facilitates bevacizumab-mediated immuno-stimulating effects, is a likely scenario. For details and references, see the main text.

Another promising approach is the combination of H-1PV with bevacizumab (Avastin^®^). This co-treatment was evaluated in a series of compassionate virus uses in recurrent glioblastoma patients. Bevacizumab is an anti-VEGF-A monoclonal antibody available in Europe since 2005 for the treatment of breast, lung, kidney, colon, ovarian and endometrial carcinomas. In 2009, bevacizumab was approved by the Food and Drug Administration (FDA) for application in glioblastoma patients [59]. While achieving a steroid-sparing effect and alleviation of edema, bevacizumab monotherapy has, however, not demonstrated significant survival benefits [60]. On the other hand, scientists and clinicians have gathered an extensive—and yet to grow—knowledge of bevacizumab’s mode of action. In particular, bevacizumab was found to exert immunomodulating activity by counteracting VEGF-induced negative effects on DC maturation, antigen presentation and lymphocytic trafficking [61]. These bevacizumab properties, together with the ParvOryx01 trial experience showing H-1PV treatment-associated immunogenic changes in glioblastoma TME, have opened up prospects for novel anti-glioma combinatorial immunotherapy development, i.e., H-1PV + bevacizumab (Figure 3). A compassionate use proof-of-concept program was conducted in five GBM patients, who developed a second or third recurrence after being treated in the ParvOryx01 trial. The patients underwent tumor resection, followed by local H-1PV administration and bevacizumab. The mean survival after treatment was extended to 15.4 months. Moreover, in three out of the five patients, striking remission of the recurrence was observed, providing first clinical hints of synergistic glioblastoma suppression through parvoviro-immunotherapy [62].

### 3.3. H-1PV + PD-1 Immune Checkpoint Inhibitors (Nivolumab)

Checkpoint blockade, a strategy which aims at overcoming immune system tolerance towards the tumor through the release from negative regulators of immune activation (immune checkpoints), is presently at the leading edge of cancer immunotherapy. Although efficient in controlling various other solid tumors, immune checkpoint inhibitors (ICIs) frequently fail to achieve a significant response in glioblastoma patients [63]. Several preclinical studies and clinical trials have therefore been initiated, in order to determine the optimal ICI-based combinations and redefine the future standards of care for this deadly disease [64].

First clinical hints of improved antitumor effects of H-1PV virotherapy upon combination with checkpoint blockade were obtained through compassionate virus uses. A series of three patients with rapidly progressing recurrent glioblastoma were treated with H-1PV (two were irradiated prior to virus administration), followed by bevacizumab and the programmed cell death protein 1 (PD-1) inhibitor nivolumab. In addition, all patients received the HDACi VPA. This innovative PV-based multimodal strategy led to radiologically confirmed tumor regression accompanied by clinical improvement in all subjects 4 to 8 weeks after virus injection [65]. An objective tumor response was also seen in another group of primary or recurrent glioblastoma patients, who received H-1PV in combination with bevacizumab and checkpoint blockade. Complete to partial tumor remission was documented in 78% of the cases, which is a significantly higher response rate than the one reported in the literature for bevacizumab- and ICI-based monotherapies [66].

Altogether, the above data provide a strong impetus for further clinical development of H-1PV combinations with radiation and/or immunomodulators (in particular bevacizumab and ICIs) in the fight against glioblastoma.

## 4. Parvovirus-Based Combinatorial Immunotherapy against Colorectal Cancer

CRC is another major cause of cancer-related deaths worldwide. Although the implementation of early-detection screening programs has substantially improved the 5-year overall survival, prognosis for CRC patients with stage 4 metastatic disease remains poor [67]. Immunotherapy, in particular checkpoint blockade, has proved efficient against heavily mutated colorectal tumors. However, it fails to elicit sufficiently strong therapeutic responses in carcinomas, which are mismatch-repair-proficient (pMMR) and possess low levels of microsatellite instability (MSI-L). Low mutational burden, together with the lack of immune cell infiltration, contribute to pMMR-MSI-L immune resistance [68]. Novel approaches are therefore needed for the treatment of patients with advanced metastatic or low mutational burden CRC. One such approach, combinatorial immunotherapy, holds much potential for extending the scope of checkpoint blockade so as to bring benefit also to CRC patients with unfavorable prognosis.

### H-1PV + CTLA-4 Immune Checkpoint Blockade (Tremelimumab)

Many tumor types, including CRC, overexpress the immune checkpoint cytotoxic T-lymphocyte-associated protein 4 (CTLA-4) and thus transmit inhibitory signals to T cells [69]. This immune evasion strategy creates an immunosuppressive environment, which allows the tumor to escape immune recognition and destruction. The anticancer effects of tremelimumab, a CTLA-4-specific human antibody, applied either alone or in combination with H-1PV, were studied by Heinrich et al. [70] n a human in vitro CRC model. H-1PV infection alone was found to reduce the viability of SW480 CRC cells and enhance extracellular CTLA-4 expression. SW480 cells and immature DCs (iDCs) co-culture experiments demonstrated that the expression of DC maturation and activation markers, namely CD83, CD80 and CD86, sharply increased when the tumor cells were infected with H-1PV. Notably, additional treatment of H-1PV-infected SW480 cells with tremelimumab resulted in IFN-γ enrichment of the co-culture supernatant [70].

## 5. Parvovirus-Based Combinatorial Immunotherapy against Melanoma

Cutaneous melanoma, also known as black skin cancer, is an aggressive tumor arising from the melanocytes. Over the past 10 years, melanoma has become a prototype for testing novel targeted therapies, first and foremost, immune checkpoint blockade. PD-1 inhibition has shown significant clinical success in controlling locoregional melanoma [71]. However, metastatic melanoma is a severe life-threatening condition for which reinforcement of current treatment tools and approaches is still needed.

### H-1PV + CTLA-4 (Ipilimumab)/PD-1 (Nivolumab) Immune Checkpoint Blockade

In order to investigate the immunological effects of H-1PV in combination with ipilimumab and/or nivolumab, a human ex vivo melanoma model was used by Goepfert et al. Similar to the observations made in CRC-derived cells [70], upregulation of immune checkpoints, CTLA-4, PD-1 and PD-L1 in particular, was seen in H-1PV-infected melanoma cells. Yet, the virus potentiated the capacity of melanoma cells to induce iDC maturation in co-culture experiments. Nivolumab and ipilimumab, when added to the treatment scheme, triggered a further increase in the release into the co-culture supernatant of IFN-γ and TNF-α, respectively. Further to this, upon combination with H-1PV, the two ICIs induced stronger CTL activation, compared to virus alone [72]. Combining PV-induced immunogenic oncolysis with CTLA-4 and/or PD-1 blockade allows achieving a double goal, i.e., tumor cell killing and activation of the immune system against the tumor. This triggering of complementary events, centered on tumor destruction and immune-mediated elimination, renders the H-1PV + ICI approach promising for melanoma and other solid tumors’ treatment.

## 6. Conclusions

Preclinical research and clinical experience have demonstrated the multimodal anticancer activity of the oncolytic parvovirus H-1PV. Two essential facets of H-1PV-induced tumor suppression consist of direct killing of malignant cells (oncolysis) and activation of cellular immune responses against the tumor. H-1PV infection, oncolysis and immune stimulation are interconnected, coordinated events, which cooperate towards multisided tumor elimination.

**Figure 4:**
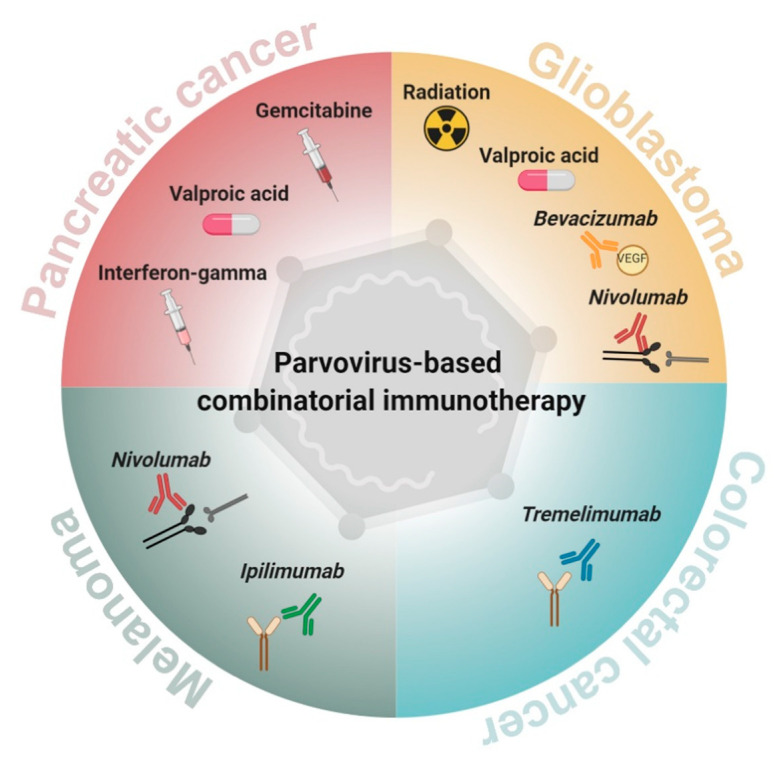
H-1PV inclusion into combinatorial anticancer immunotherapy regimens. The development of H-1PV combinations with ionizing radiation, chemotherapeutics, histone deacetylase inhibitors (HDACis), angiogenesis inhibitors and immunomodulators holds significant promise for the future of poor-prognosis solid cancer treatment.

Glioblastoma and pancreatic adenocarcinoma are among the most devastating human malignancies, characterized by resistance to current therapies, tendency to recurrence and an overall poor outcome. H-1PV has undergone clinical testing in two recently conducted trials, ParvOryx01 in glioblastoma and ParvOryx02 in pancreatic carcinoma. Virus excellent safety and tolerability, together with the capacity for gentle TME immune landscape proinflammatory modulation, provide a strong impetus for further H-1PV clinical development. It should, however, be noted that in the clinical setting, various patient-dependent factors may result in suboptimal antitumor effects. Large intra-tumoral tissue heterogeneity, emergence of tumor cells resistant to virus infection/killing, dominance of the highly immunosuppressive TME, hampered virus spreading, off-target infection and virus neutralization by antiviral antibodies are among the major barriers to efficient H-1PV-induced tumor elimination. While various other approaches (capsid modification, chimera generation, fitness mutant selection, armed vector construction) to H-1PV treatment optimization are currently under investigation, PV-based combinatorial therapies are considered as a particularly promising avenue that holds the potential of enhancing both oncolysis and immune-mediated tumor destruction. Combinations of the virus with other anticancer approaches, namely irradiation, chemotherapy (gemcitabine), epigenetic modulation (HDACi), angiogenesis regulation (bevacizumab) or immunotherapy (immune checkpoint blockade), were evaluated in both preclinical models and in cancer patients. The combinatorial H-1PV-based viro(immuno)therapeutic strategy was proven to achieve greater anticancer effects compared to individual agents alone. The synergistic boost was particularly pronounced in combinations including the HDACi VPA, bevacizumab or the PD-1 inhibitor nivolumab. Glioblastoma patients treated with this combination showed striking tumor remission and extended survival, notably after second or even third recurrence. These early clinical observations speak in favor of considering H-1PV inclusion into various immunotherapeutic protocols against glioblastoma and other poor-prognosis solid tumors (Figure 4).
